# Effects of Animal, Climatic, Hunting and Handling Conditions on the Hygienic Characteristics of Hunted Roe Doer (*Caprelous capreolus* L.)

**DOI:** 10.3390/foods9081076

**Published:** 2020-08-07

**Authors:** Raffaella Branciari, Andrea Onofri, Fausto Cambiotti, David Ranucci

**Affiliations:** 1 Department of Veterinary Medicine, University of Perugia, 4 San Costanzo Street, 06126 Perugia, Italy; raffaella.branciari@unipg.it; 2 Department of Agriculture, Food and Environmental Sciences, University of Perugia, 74 Borgo XX Giugno, 06126 Perugia, Italy; andrea.onofri@unipg.it; 3 Health Department Umbria 1-Alto Chiascio, 38 Cavour Street, 06024 Gubbio (PG), Italy; fausto.cambiotti@uslumbria1.it

**Keywords:** game meat, aerobic colony count, enterobacteriaceae, food safety, *salmonella*

## Abstract

The population of wild animals is increasing, and control strategies based on selective hunting are among the major options adopted. The game meat obtained is therefore available for controlled and certified valuable chains. The understanding of carcass contamination and the factors affecting it is therefore crucial to ensure meat safety and prolonged shelf-life. The carcass hygiene of 64 hunted wild male roe deer (*Capreolus capreolus* L.) was evaluated in relation to factors potentially affecting it. Aerobic colony and *Enterobacteriaceae* counts, as well as *Salmonella* spp. and *Listeria monocytogenes* detection, were performed. The interaction of the microbial determination with age and weight of the animals, the climate conditions, the shooting procedure, the time between the killing and the evisceration as well as the time of storage of the carcasses in refrigerated conditions before skinning, were evaluated. Neither *Salmonella* spp. nor *Listeria monocytogenes* were detected on the carcasses and the average loads detected were 3.39 ± 1.06 UFC/cm^2^ and 2.27± 1.11 UFC/cm^2^ for the aerobic colony count and *Enterobacteriaceae* count, respectively. The loads detected are similar to those reported by UE legislation for slaughtered species. The time of storage before skinning, the environmental temperature during hunting and the time between shooting and evisceration, associated with animal weight, affect the carcass hygiene and must be taken into careful consideration by hunters as food business operators.

## 1. Introduction

The increase in the wild animal population in Europe has revealed some issues related to the growing relationship between wild ungulates and man-made environment and animal protection [1]. Among these issues, the risks for livestock and human health, the damages to crop production and the car collisions are good examples [2,3]. To counteract these phenomena, among the most implemented strategies is wild ungulate monitoring followed by population control based on specific hunting periods for targeted animals, performed by trained hunters [4,5]. Nonetheless, the game meat obtained through these control measures is a relevant food source that could be exploited for modern consumers [6] who search for “green” and healthier products [7]. Roe deer (*Capreolus capreolus* L. 1774) is one of the wild species that in the last decade has been increasing in central Italy, and specific hunting seasons for its control are annually set. The meat obtained could be directly consumed by hunters, but according to European legislation [8], small quantities of these game meats could be sold directly to consumers by the hunters, often starting an “unregulated” market without proper safety assurance and traceability. Thus, valuable certified meat or meat product chains could be implemented as a supply for local producers or restaurants [9,10]. These certified chains have to fulfill the complete traceability of the meat and be obtained from healthy animals, and the proper hygiene of the products should be obtained by adopting good manufacturing practices during all the steps of the chain, starting from the choice of the animal to the distribution of game meat. 

The aim of the study was to evaluate the contamination of roe deer carcasses obtained during animal control plans performed in Central Italy and the effects of a series of factors such as animal features (age and weight), environmental conditions (temperature), hunting practices and the amount of time it takes to carry out the main recovery, bleeding, evisceration and storage procedures before the carcasses are transferred to a cutting center.

## 2. Materials and Methods 

### 2.1. Hunting Remarks and Carcass Sampling

The trial was conducted during 2 hunting seasons (2018 and 2019) performed for the control of the roe deer population in Gubbio (Umbria Region, Central Italy). The hunting area was near (about 2 km) a collection center specifically designed for wild ungulates storage where carcasses could be weighted and promptly refrigerated after evisceration. A specific survey questionnaire was drawn up and agreed with the hunter who had to complete it during hunting season. Selected answers were aimed at most of the defined questions (Table 1). Only one qualified hunter was enrolled to ensure continuity in the hunting practice. The selected hunter was trained in an official course for the best practice during hunting procedures. The hunting technique used did not include dogs but consisted in shooting the animals that passed near the hidden waiting station. This also allowed for a selection of the hunted animals and an accurate evaluation of their health statuses (i.e., showing abnormal behaviors, extremely thin or cachexic animals, presenting bodily impairment or abnormal secretions from orifices). The hunting was performed during early mornings or evenings with sufficient natural light. The procedure was conducted using a rifled gun and no-lead ammunitions. The hunter was trained to not shoot the animal in the abdomen and to kill it using only one shot. After the shooting and recovery of the roe deer, the animals were traced with a numbered plastic clamp fixed on the hind leg and, bled on the field; all the procedures were the same for each animal and performed by the same operator (the hunter himself). The carcasses were transferred with cleaned containers and, after the arrival to the collection center, they were weighted to obtain the carcass weight before and after evisceration. The evisceration was hygienically conducted on carcasses hung by the hind legs and the complete removal of the thorax and abdominal viscera was performed. The person in charge of this task was trained in evisceration techniques to be adopted and not to punch or cut the gastro-intestinal tract to avoid carcass contamination. The viscera, traced with the same number as that of the plastic clamp, was checked by the qualified hunters for gross pathology after evisceration and, only if suspected lesions were present, they were then sent to the veterinarian’s office at the game-handling establishment. No lesions were detected that could cause the exclusion of the meat from human consumption. The storage of the carcasses in the collection center was performed without skinning, according to the UE legislation, for 2, 4 or 6 days at 5 ± 1 °C and then transferred to the local game-handling establishment with an authorized refrigerated truck under hygienic conditions. A total of 64 male roe deer were considered in the trial. Upon the arrival at the game-handling establishment, they were skinned, and no decontamination strategies were adopted during this procedure. Samples were collected from the carcasses with reference to destructive methods to evaluate surface microbial loads [11]. Four tissue samples of 5 cm^2^ each were obtained from four different parts: hind leg (rump), flank, brisket and foreleg. The four tissue fragments were pooled in a sterile stomacher bag (sample of 20 cm^2^) and transferred to the microbiology laboratory in a refrigerated condition.

### 2.2. Microbial Analysis

Surface tissue samples were prepared and processed for aerobic colony count (ACC) and *Enterobacteriaceae* count (ENT) according to ISO 4833-1 [12] and ISO 21528-2 [13], respectively. In brief, the fragments were homogenized (Stomacher 400 circulator, Seward Ltd., Norfolk, UK) with buffered peptone water (Oxoid Ltd., Basingstoke, UK), [14], and serial fold dilutions were made. An amount of 1 mL of the selected dilutions were plated into Petri dishes and doused with warm Platre count agar (PCA, Oxoid Ltd., Basingstoke, UK) for the ACC and with Violet Bile Glucose Agar (VRBG, Oxoid Ltd., Basingstoke, UK) for ENT. After cooling, the plates were incubated under aerobic conditions at 30 °C for 24 h and 37 °C for 48 h for ACC and ENT, respectively. Results were obtained as Colony Forming Units (CFU)/cm^2^ and transformed into logarithmic values. Salmonella spp. isolation was performed according to ISO 6579-1 [15] with samples per-incubated with buffered peptone water for 24 h. An amount of 0.1 mL of the homogenates was transferred into Rappaport Vassiliadis Soya peptonebroth (RVS, Oxoid Ltd., Basingstoke, UK) incubated for 24 h at 42 °C, and Muller–Kaufmann Tetrathionate-Novobiocin Broth (MKTTn, Oxoid Ltd., Basingstoke, UK) incubated for 24 h at 37 °C. A loopful of broth was plated onto Xylose-Lysine-Desoxycolate Agar (XLD, Oxoid Ltd., Basingstoke, UK) and *Salmonella* Chromogenic Agar (Oxoid Ltd., Basingstoke, UK) and incubated at 37 °C for 24 h. A second pool of samples of the four tissue fragments, obtained from the same carcass sites, were used for *Listeria monocytogenes* detection according to ISO 11290-1 [16]. After a pre-enrichment step performed in half-Fraser broth (Biolife, Milan Italy) and incubated for 24 h at 30 °C, samples were sub-cultured in Fraser broth (Biolife, Milan Italy) and incubated for 24 h at 37 °C [17]. A loopful of broth was plated onto the selective Agar Listeria Ottaviani Agosti medium (ALOA Oxoid Ltd., Basingstoke, UK) and incubated aerobically at 37 °C for 24–48 h.

### 2.3. Statistical Analysis

The whole dataset was submitted to separate statistical analyses for each of the two response variables on a logarithmic scale (ACC and ENT). ACC and ENT were considered as dependent variables and the other variables (environmental temperature, animal age and weight, shooting condition, hunting condition and storage period inside the collection center cell) as independent factors. For each variable, 9 predictors were considered as the experimental factors; initially, the significance of the effect of each single factor on each of the response variables was assessed by using one-way ANOVA. Adjusted means (least square means) were derived from the models and compared by using Tukey HSD. Secondly, the factors which showed significance in ”univariate” analyses were used to fit a multi-way additive ANOVA model in a stepwise forward fashion, aimed at discovering which variables produced the most relevant effect, based on the Akaike Information Criterion (AIC) [18].

## 3. Results

Carcasses of 64 male roe deer were collected at different conditions—each reported in Table 2. The temperature during the hunting period ranged between 4 and 26 °C (average value: 14.1 °C ± 6.03 standard deviation). All the subjects were in good condition and shot by a rifled bore shotgun with a single no-lead bullet. Only one shot was performed to kill the animals and no shots were fired in the abdomen of the subjects. The animals’ average weight was 22.79 kg (standard deviation = 3.78) and the average age was 2.41 years (standard deviation = 0.91). The classes recorded in the questionnaires about hunting conditions were processed to have the total time elapsed from the moment the shot was discharged to the individual operations carried out. The subjects were recovered always before 60 min from the shot, bled out in a field for 90 min, eviscerated within 4 h and promptly refrigerated. No rupture of the intestines was registered neither due to the shot nor to the evisceration procedures adopted. 

**Figure 1:**
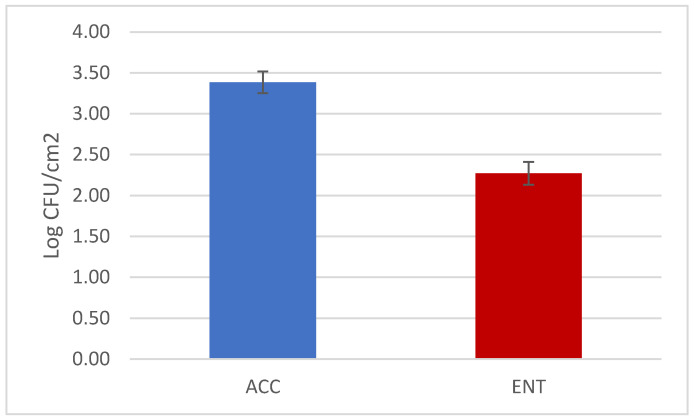
Results of the aerobic colony count (ACC) and *Enterobacteriaceae* count (ENT) of the roe deer carcasses (mean values and standard errors).

**Figure 2:**
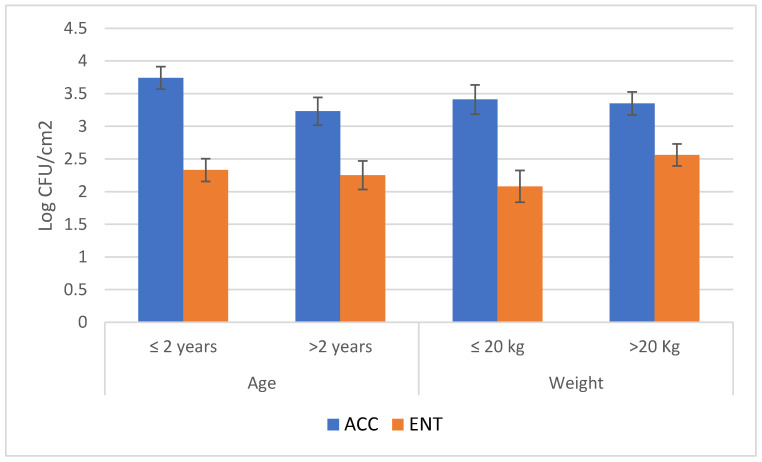
Results of the microbial loads of roe deer carcasses (aerobic colony count-ACC and *Enterobacteriaceae* count-ENT) according to the animal characteristics (mean values and standard errors). ACC = aerobic colony count; ENT = *Enterobacteriaceae* count.

**Figure 3:**
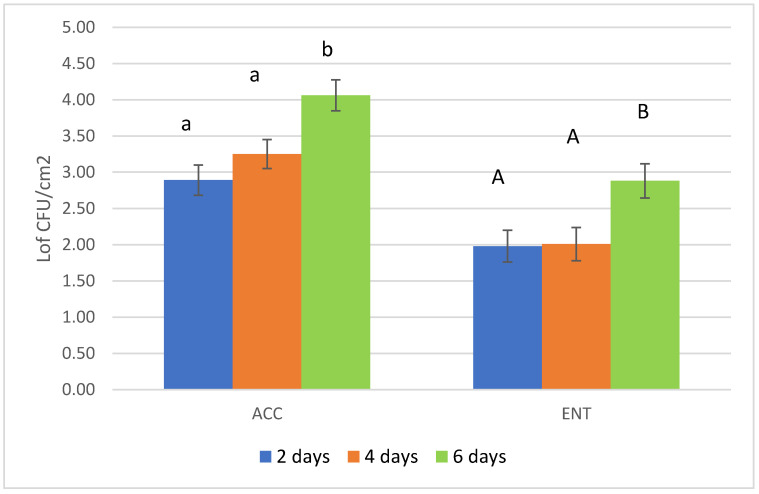
Results of the microbial loads (aerobic colony count-ACC and Enterobacteriaceae count-ENT) according to the length of the storage period before skinning of roe deer carcasses. ACC = aerobic colony count; ENT = *Enterobacteriaceae* count. Different letters represent a significant difference in the mean values reported for each parameter considered (*p* < 0.05).

The overall results of the loads are reported in Figure 1. The mean values were below 3.5 and 2.5 Log CFU/cm^2^ for ACC and ENT, respectively. Considering the hunting questionnaires, it is possible to see that several variables, when considered independently from one another, had significant effects on the microbial load. These variables represented the time elapsed between the shot and evisceration and storage time before skinning for ACC and environmental temperature and storage of the carcasses for ENT (Table 3 and Table 4, Figure 2 and Figure 3). The multifactorial analyses reveal that the most relevant effect for the carcass hygiene was due to storage length before the skinning, which caused the highest impact on the AIC value, both for ACC and ENT. 

On the same basis, the animal weight is the second factor that must be considered for ACC, while the environment temperature is the second factor for ENT. The other factors were negligible in this study.

The detection of the selected foodborne pathogens from the surface of the carcasses was always negative as neither *Salmonella* spp. nor *Listeria monocytogenes* were isolated. 

## 4. Discussions

The ACC and ENT mean values recovered from the surface samples were similar to those reported by Avagnina et al. [19], in roe deer hunted in the Italian alps region; by Klupsaite et al. [20] in Lithuania; and by Atanassova et al. [21] in Germany. Similar ACC values were obtained in the game meat of other species even outside Europe [22,23]. Furthermore, Membré et al. [24] report a level of 2.37 Log CFU/cm^2^ *Enterobacteriaceae* count on roe deer meat cuts. The AAC and ENT values fall into the hygienic criteria limit set by the EU commission for slaughtered carcasses at abattoirs [11], and only three samples were at an unsatisfactory level for ACC and eight for ENT. Despite these criteria, which are implemented to evaluate the slaughtering hygiene during a period of time and pools of different carcasses during the same day taken into consideration in the EC Regulation [11], these findings confirm that the procedures adopted during hunting on the carcasses, if properly and hygienically implemented, could be as hygienic as at the slaughterhouse level [19,25,26]. Furthermore, the results registered are close to those of slaughtered animals, such as ovine [27,28].

Regarding the single effect, different authors highlight the role of the environmental condition during hunting on the hygienic characteristics of game meat, but no data are available yet on roe deer carcasses. Stella et al. [29] report a decrease in microbial load on hunted wild boar carcasses when the environmental temperature was above 10 °C, which confirms the similar trend found for ENT in the present trial but not for ACC. The high temperature could be responsible for a delay in chilling the carcasses and therefore an increase in microbial growth [30].

No difference was detected among the age and weight groups. Both classes could influence the general carcass hygiene, as the ungulates’ weight is generally associated with their age, and processes performed on heavier animals are generally more laborious [29]. These conditions are more evident in animals with high sexual dimorphisms, such as wild boar, with males that are heavier than females with the increase in their age [29]. Nonetheless, the multivariate analysis indicates the weight as the second factor, after the period of storage before skinning, affecting the ACC count of the roe deer carcasses. 

Among the hunting conditions, the only statistically different effect was the time between the shot and evisceration of the roe deer and only for ACC. This effect is suggested by different authors both in roe deer and wild boar [19,31], as the shorter the elapsed time for carcass evisceration the lower the risk of microbial spread and spoilage [30]. Nonetheless, the hunting procedures considered in this research were relatively short and always under 4 h. Some authors considered the best procedure to eviscerate hunted ungulates within 3 h of the shot and that only longer times are at risk [30,31]. The factor, however, does not appear significant in the AIC analysis and, therefore, could be considered marginal in roe deer. Moreover, the evisceration procedure was conducted without perforation of the intestines that could influence the final microbial loads on the carcasses.

The ammunition caliber used was not able to affect carcass hygiene as the damage caused by the shot was limited. Other kinds of ammunitions could induce large damage to the carcass and a higher extent of contamination [32,33]. Carcass damages are usually considered a relevant source of contamination, not only for ammunition type but also the number of shots, their accuracy and eventual dog bites [34,35]. Different authors also report a direct effect of abdomen hitting on game meat hygiene, especially for ENT [23,25,36], but there is no agreement on this topic [37]. The hunting procedures adopted in the present trial were well performed as no multiple shot was needed to kill the animals, no abdomen was hit and no dogs were used during hunting. Nonetheless, a trend in higher ACC on the carcasses when the lower thorax region was shot must be mentioned and could be due to upper gut tract (prestomacs) spilling. 

The most important effect on carcass hygiene registered in the present survey is the extent of the storage period of the unskinned carcasses inside refrigerated cells in the collection center. After 6 days of storage, the microbial loads were statistically higher than 2 to 4 days of storage before transferring the carcass to the game-handling establishment for skinning. Authors report a higher value of ACC and ENT loads when this step is prolonged because skin and fur could increase the risk of contamination [38,39] despite the carcasses being stored in chilled chambers. These observations refer to wild boar, as no data are available for roe deer carcasses. The behavior of storing game carcasses without skinning is quite common among hunters, with the conviction of a better aging of the meat and to optimize the logistics related to their transfer to the game-handling establishment. Nonetheless, an increase in microbial loads under this storage condition inside the deep muscle tissue is reliable [40]. Therefore, a reduction in storage time and proper skinning are recommended. 

Safety is another crucial aspect in game meat, and the absence of relevant pathogens is particularly important for consumer care. No *Salmonella* spp. was detected in the sampled carcasses proving the relative low risk of deer meat for these pathogens [25,41,42]. *Salmonella* spp. is rarely observed in wild cervids in general and in roe deer meat in particular [19,43], highlighting that its possible presence is mainly due to cross-contamination during meat processing. *Listeria monocytogenes* was not detected on the samples as reported by other authors in game meat [44], and a low prevalence of positive samples in different wild ungulates is generally registered [19,25]. The presence of this pathogen on the surface of the carcasses is probably due to environmental contamination, not only from animal specimens [45] but also in the establishments, where accurate hygienic processes could reduce its prevalence. Nonetheless, this pathogen must be taken into consideration when ready-to-eat products are obtained from roe deer meat [46].

## 5. Conclusions

The present survey reveals good hygienic conditions of roe deer carcasses if the hunting procedures are carried out correctly and in a short period of time. Training hunters who carry out procedures, such as bleeding and evisceration, must be considered to prevent carcass contamination. Furthermore, when a certified supply chain is implemented, the availability of collection centres is also crucial to guarantee an excellent hygienic profile. Attention should be paid to storage time and the condition of the carcasses before skinning as a critical point in carcass contamination.

## Figures and Tables

**Table 1:** Questionnaire adopted during the hunting procedures.

Questions	Selected Answers				
**General question**					
Date and time (hour)					
Environmental temperature °C	<10 °C	10–15 °C	>15 °C		
**Roe deer generality**					
Gender	male	female			
Age (presumptive)					
Weight (after shooting) in kg					
Animal condition	good	bad			
**Shooting conditions**					
Shotgun	Smooth bore	Rifled bore			
Bullet	Single	Buckshot			
Ammunition caliber	7.0 mm	7.8 mm			
Shooting site	Head	Neck	Shoulder/ hearth	Low thorax	Abdomen
**Hunting condition**					
Time between shooting and collection	<15 min	15–29 min	30–59 min	60–90 min	
Time between collection and bleeding	<15 min	15–29 min	30–59 min	60–90 min	
Time between bleeding and evisceration	<30 min	30–59 min	60–90 min		
Rupture of the intestine	yes	no			

**Table 2:** Distribution of the roe deer carcass samples according to selected classes.

Questions	Classes			
Environmental temperature °C	<10 °C	10–15 °C	>15 °C	
numbers of samples	13	21	30	
Age (presumptive)	≤2 year	>2 year		
numbers of samples	39	25		
Weight (after shooting) in kg	10–20 kg	20–30 kg		
numbers of samples	20	44		
Ammunition caliber	7.0 mm	7.8 mm		
numbers of samples	28	36		
Shooting site	Head	Neck	Shoulder/Heart	Low Thorax
numbers of samples	18	13	24	9
Time between shooting and collection	<15 min	15–29 min	30–60 min	
numbers of samples	27	28	9	
Time between shooting and bleeding	<30 min	31–60 min	61–90 min	
numbers of samples	27	27	10	
Time between shooting and evisceration	30–59 min	1.0–2.5 h	2.5–4 h	
numbers of samples	46	11	7	
Time of storage before skinning	2 days	4 days	6 days	
numbers of samples	21	21	20	

**Table 3:** Results of the microbial loads of roe deer carcasses according to the environmental conditions (mean value ± standard error).

	Environmental Temperature			*p* Value
	<10 °C	10–15 °C	>15 °C	
ACC	3.32 ± 0.298	3.38 ± 0.234	3.42 ± 0.196	0.963
ENT	1.48 ± 0.29 ^a^	2.43 ± 0.19 ^b^	2.53 ± 0.23 ^b^	0.012

ACC = aerobic colony count; ENT = *Enterobacteriaceae* count. Different letters in the same row represent a significant difference in the mean values reported (*p* < 0.05).

**Table 4:** Results of the microbial loads of roe deer carcasses according to the hunting conditions.

	Ammunition Caliber						*p* Value
	7 mm			7.8 mm			
ACC	3.38 ± 0.202			3.39 ± 0.18			0.969
ENT	2.20 ± 0.21			2.33 ± 0.19			0.641
**Shooting point**							
	*Head*	*Neck*		*Heart*		*Thorax*	
ACC	3.14 ± 0.21	3.21 ± 0.21		3.52 ± 0.25		4.01± 0.35	0.164
ENT	2.43 ± 0.27	2.07 ± 0.31		2.23 ± 0.23		2.34 ± 0.38	0.841
**Time between shooting and collection**							
	<15 min		15–29 min		30–60 min		
ACC	3.25 ± 0.20		3.31 ± 0.19		4.02 ± 0.35		0.151
ENT	2.23 ± 0.22		2.29 ± 0.21		2.33 ± 0.38		0.969
**Time between shooting and bleeding**							
	<30 min		31–60 min		61–90 min		
ACC	3.25 ± 0.20		3.27± 0.20		4.04± 0.33		0.098
ENT	2.23 ± 0.22		2.28 ± 0.22		2.36 ± 0.36		0.955
**Time between shooting and evisceration**							
	30–59 min		1.0–2.5 h		2.5–4 h		
ACC	3.19 ± 0.151 ^a^		3.73 ± 0.309 ^a,b^		4.10 ± 0.387 ^b^		0.049
ENT	2.24 ± 0.17		2.26 ± 0.34		2.51 ± 0.43		0.840

ACC = aerobic colony count; ENT = *Enterobacteriaceae* count. Different letters in the same row represent a significant difference in the mean values reported (*p* < 0.05).
